# Factors Associated With the Improvement of Left Ventricular Systolic Function by Continuous Positive Airway Pressure Therapy in Patients With Heart Failure With Reduced Ejection Fraction and Obstructive Sleep Apnea

**DOI:** 10.3389/fneur.2022.781054

**Published:** 2022-03-10

**Authors:** Ryo Naito, Takatoshi Kasai, Tomotaka Dohi, Hisashi Takaya, Koji Narui, Shin-ichi Momomura

**Affiliations:** ^1^Department of Cardiovascular Biology and Medicine, Juntendo University Graduate School of Medicine, Tokyo, Japan; ^2^Cardiovascular Respiratory Sleep Medicine, Juntendo University Graduate School of Medicine, Tokyo, Japan; ^3^Sleep Center, Toranomon Hospital, Tokyo, Japan; ^4^Department of Medicine, Saitama Citizens Medical Center, Saitama, Japan

**Keywords:** CPAP, OSA (obstructive sleep apnea), short-term, HFrEF (heart failure with reduced ejection fraction), LVEF (left ventricular ejection fraction)

## Abstract

**Background:**

Obstructive sleep apnea (OSA) is a potential risk factor in cardiovascular diseases, including arrhythmia, coronary artery disease, and heart failure (HF). Continuous positive airway pressure (CPAP) therapy is an effective therapy for OSA and the underlying HF, partly through a 5–9% increase in the left ventricular ejection fraction (LVEF). However, the data on the factors associated with the efficacy of CPAP on LVEF in patients with HF complicated by OSA are scarce. This study aimed to investigate whether LVEF improves in patients with OSA and HF after 1 month of CPAP therapy, and to clarify which factors are associated with the degree of LVEF improvement.

**Method:**

This was a prospective, single-arm, open-label study. We enrolled moderate-to-severe patients with OSA and HF who were being followed up at the cardiovascular center of Toranomon Hospital (Tokyo, Japan). The parameters of sleep study and LVEF were assessed at the baseline and after 1 month of CPAP. The multivariate regression analyses, with changes in LVEF as a dependent variable, were performed to determine the factors that were associated with the degree of LVEF improvement.

**Results:**

We analyzed 55 consecutive patients with OSA and HF (mean age: 60.7 ± 12.2 years, mean LVEF value: 37.2 ± 9.8%). One month of CPAP treatment decreased the apnea-hypopnea index (AHI) from 45.3 ± 16.1 to 5.4 ± 4.1 per hour, and the LVEF improved from 37.2 ± 9.8 to 43.2 ± 11.7%. The multivariate regression analyses demonstrated that age and body mass index (BMI) were significant determinants of LVEF improvement.

**Conclusion:**

The LVEF improved significantly after 1 month of CPAP therapy in Japanese patients with OSA and HF. Multivariate regression analyses indicated that an improvement in LVEF was likely to be observed in young patients with obesity.

## Introduction

The aging of the population and prolongation of the lives of patients with cardiovascular disease due to improvements in therapy have resulted in an increased incidence of HF (1). Despite advances in both pharmacologic and non-pharmacologic therapies, the mortality and the rate of repeat hospitalization due to exacerbation of HF remain high (2, 3). Therefore, further treatment strategies targeting conditions with potential HF risk are needed.

Obstructive sleep apnea (OSA), one of the most prevalent sleep-disordered breathing characterized by repeated episodes of upper airway obstruction during sleep, consequent chronic intermittent hypoxia, and sleep fragmentation, has been recognized as a potential risk factor in the development of cardiovascular diseases (4–6). The Sleep Heart Health Study showed an adjusted hazard ratio for incident HF of 1.58 for men with AHI of ≥30 compared with those with AHI of <5 (7). The prevalence of OSA in HF populations is reportedly high (4, 8, 9). An observational study reported a negative prognostic impact of untreated OSA in patients with HF (10).

Based on the results of clinical studies, CPAP therapy has been demonstrated as an effective therapy for OSA and the underlying HF (10, 11) through an increase in the LVEF (12, 13). The degree of improvement in LVEF varied from 5 to 9% in these studies (12, 13). However, no study has investigated the efficacy of CPAP for OSA and underlying HF among the Japanese population. Moreover, it is unclear which factors are associated with the degree of LVEF improvement by CPAP therapy. This study aimed to investigate whether LVEF in Japanese patients with OSA and HF improved after 1 month of CPAP therapy and to clarify which factors are associated with the degree of LVEF improvement.

## Methods

### Trial Design, Population, and Study Period

We enrolled patients who were being followed up at the cardiovascular center in Toranomon Hospital (Tokyo, Japan) between January 1, 2001 and March 1, 2005, if they met the following inclusion criteria: (1) the presence of symptomatic HF with reduced LVEF, which was defined as an LVEF of <50% on echocardiography (14) within 1 month before the diagnostic sleep study, and with New York Heart Association (NYHA) Class II or above; (2) stable clinical status, defined as the absence of hospital admissions and obtainment of optimal medical therapy for at least 1 month before enrollment in the study; (3) having a diagnosis of moderate-to-severe sleep apnea from a sleep study, which was defined as ≥ 15 apnea or hypopnea events per hour of sleep (i.e., AHI); and (4) good adherence to CPAP (night usage of ≥ 4 h on 70% of the days during CPAP therapy) (15) during the initial month. The exclusion criteria were as follows: (1) age <20 or > 80 years, (2) the presence of known untreated neoplasms, (3) a history of stroke with neurologic deficit, and (4) a history of severe chronic pulmonary disease. Informed consent was obtained from all the patients who participated in the study. The study was conducted in compliance with the Declaration of Helsinki and in accordance with the ethics policies of the institutions involved.

### Sleep Study and CPAP

All patients were diagnosed with sleep apnea based on the results of overnight polysomnography (PSG) using a digital polygraph (SomnoStarα Sleep System; SensorMedics Corp.; Yorba Linda, CA, USA) at our sleep laboratory. We used the definitions and scoring methods for sleep apnea (16, 17). We defined patients with predominantly central sleep apnea as having an AHI of ≥15 events/h of sleep, of which > 50% were central events. These patients were excluded from further analyses. The remaining patients who were diagnosed with moderate-to-severe OSA received CPAP therapy. The CPAP was titrated manually during a second overnight sleep study to determine the appropriate pressure level for each patient. Thereafter, a CPAP therapy without supplemental oxygen was initiated for the patients. The patients were instructed to use the device while sleeping at home.

### Measurements

Body mass index (BMI), systolic and diastolic blood pressure (BP), heart rate (HR), and subjective sleepiness assessed by the Epworth Sleepiness Scale (ESS) (18) were assessed at the baseline and 1 month later. Left ventricular systolic function was expressed as LVEF, and the plasma norepinephrine level and NYHA functional classes were evaluated at the baseline and 1 month later. The LVEF was measured by echocardiography using the modified Simpson's rule, and blood samples were obtained early in the morning after the sleep study.

### Statistical Analysis

All variables are shown as mean ± SD. The comparison between parameters in diagnostic PSG and CPAP was performed using a two-tailed paired *t*-test. Changes in cardiac function were assessed using a two-tailed paired *t*-test. Univariate regression analyses were performed to determine which factors were associated with the degree of LVEF improvement, in which changes in LVEF were included as a dependent variable. The variables included in the univariate analyses were included in a stepwise multivariate regression analysis. Statistical significance was set at *p* < 0.05. Statistical analyses were performed using the statistical package for social sciences (SPSS) (SPSS Inc., Chicago, IL, USA).

## Results

**Figure 1:**
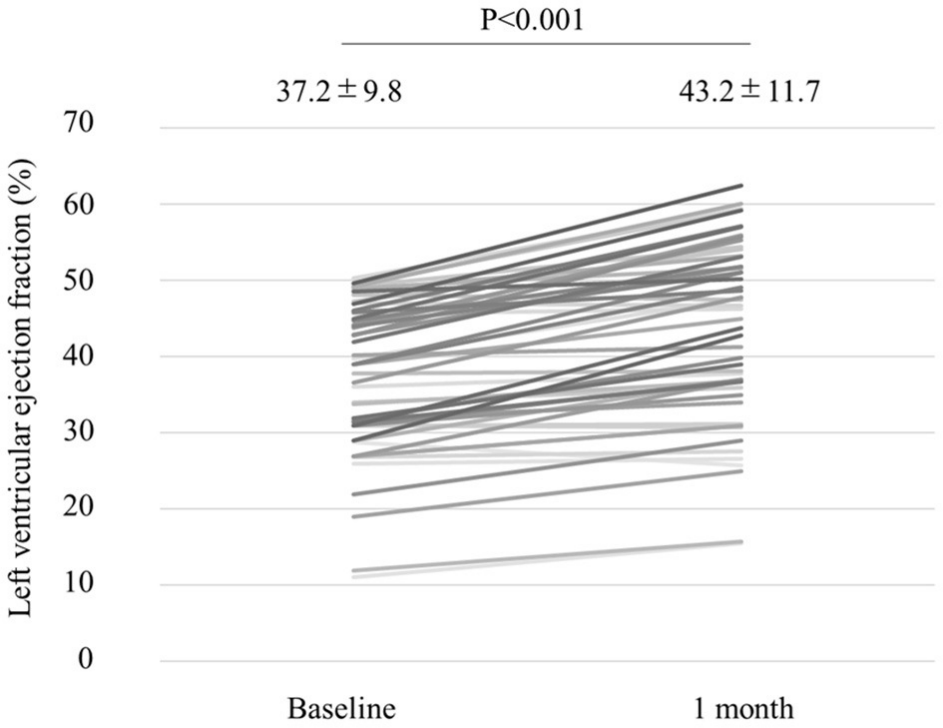
Left ventricular ejection fraction (LVEF) improved from 37.2 to 43.2%, with a statistical significance after 1 month of CPAP treatment.

**Figure 2:**
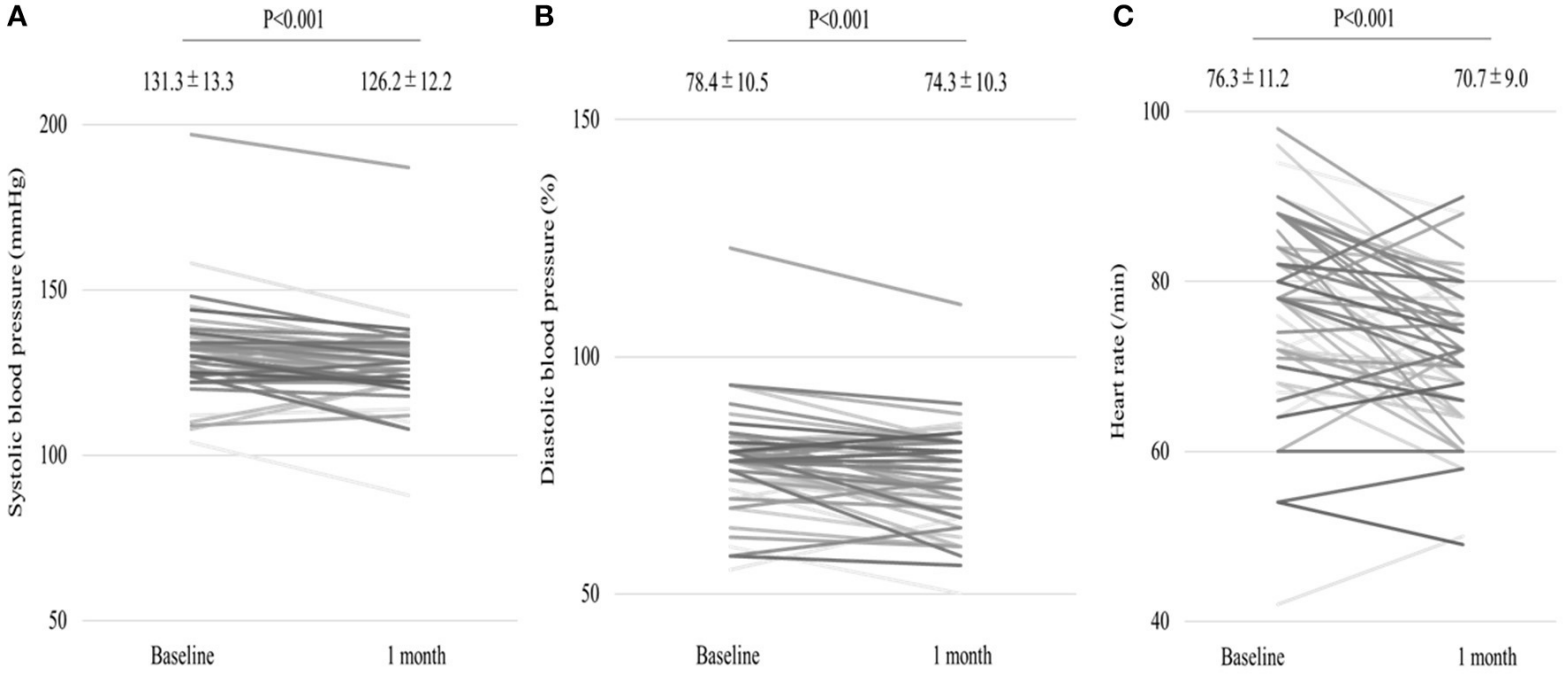
**(A,B)** Significant decrease in systolic and diastolic blood pressures (BP) as well as heart rate **(C)** after 1 month of continuous positive airway pressure (CPAP) treatment.

**Figure 3:**
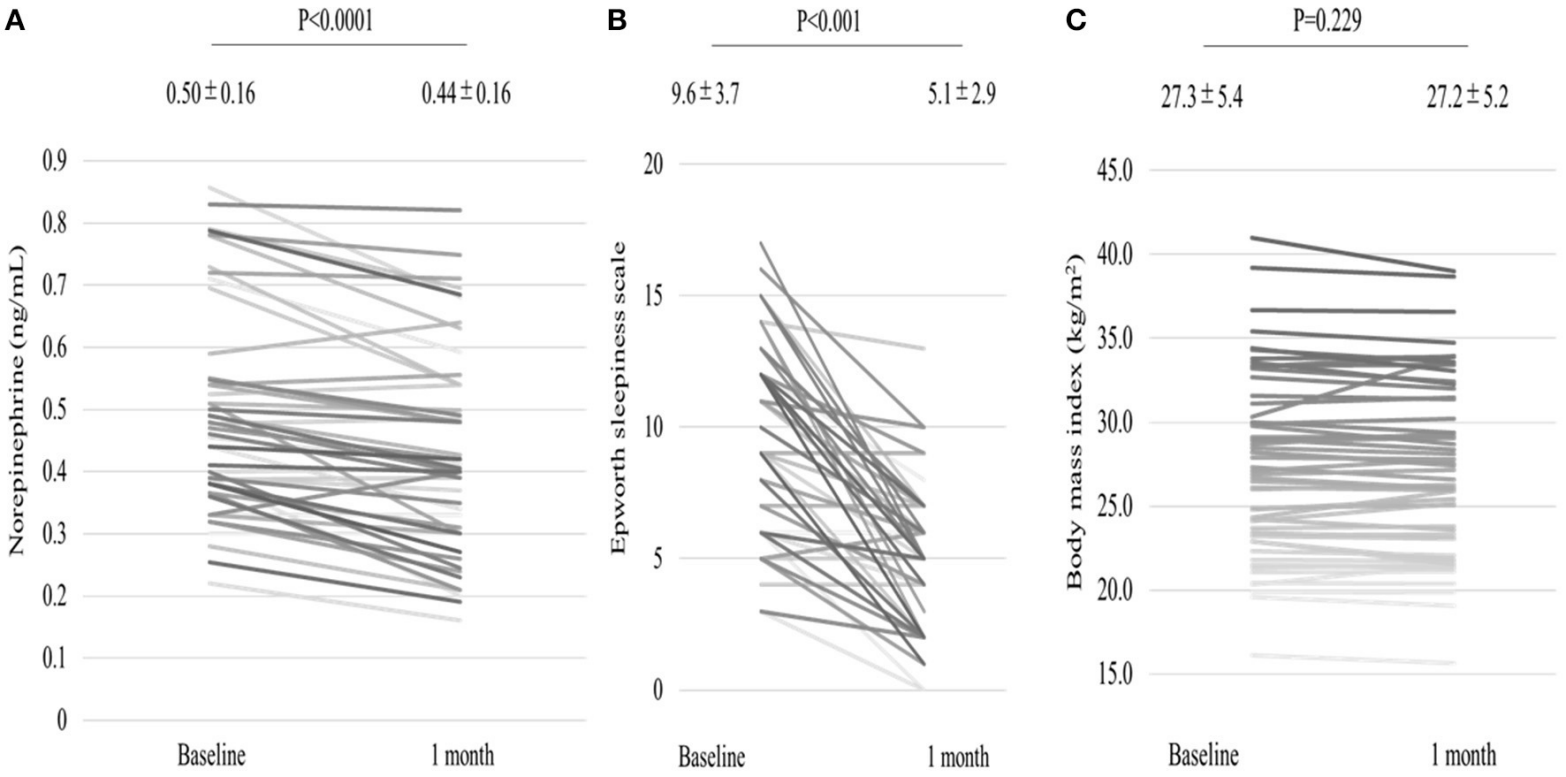
Serum norepinephrine concentration levels **(A)** decreased, and Epworth Sleepiness Scale **(B)** improved after 1 month of CPAP treatment, while no significant change in BMI **(C)** was observed.

We analyzed 55 consecutive patients with HF and reduced LVEF. The baseline patient characteristics are shown in Table 1. The proportion of men was 94.5%. The mean age was 60.7 ± 12.2 years, and the mean BMI was 27.4 ± 5.4 kg/m (2). The mean LVEF was 37.2 ± 9.8%. The data of the sleep study on diagnosis and CPAP are shown in Table 2. The data on CPAP demonstrated considerable improvements in sleep apnea and sleep quality, which were expressed as sleep stages, percentage of slow-wave sleep, and rapid eye movement (REM) sleep. The CPAP therapy decreased AHI, as well as the arousal index from 45.3 ± 16.1 to 5.4 ± 4.1 per hour and 43.9 ± 19.6 to 15.7 ± 10.3 per hour, respectively. The LVEF improved from 37.2 ± 9.8 to 43.2 ± 11.7% after 1 month of CPAP treatment (Figure 1). Significant decreases in systolic and diastolic BP and HR (Figures 2A–C) after treatment were noted. The serum norepinephrine concentration decreased, and the ESS improved significantly after the treatment, while no significant changes were observed in BMI (Figures 3A–C).

**Table 1:** Patients' characteristics.

**Variables**	**N = 55**
Age, years	60.7 ± 12.2
Male gender n (%)	52 (94.5)
Body mass index kg/m^2^	27.4 ± 5.4
Systolic blood pressure mmHg	131.1 ± 13.3
Diastolic blood pressure mmHg	78.4 ± 10.5
Heart rate /min	76.3 ± 11.2
Left ventricular ejection fraction %	37.2 ± 9.8
Norepinephrine ng/ml	0.50 ± 0.16
NYHA class	
II n (%)	32 (58.2)
III n (%)	23 (41.8)
Ischemic heart disease n (%)	12 (21.8)
Atrial fibrillation n (%)	16 (29.1)
Epworth sleepiness scale	9.6 ± 3.7
Medications	
ACE inhibitors / AR blockers n (%)	46 (83.6)
Beta blockers n (%)	33 (60.0)
Diuretics n (%)	47 (85.5)
Spironoractone n (%)	11 (20.0)
Digoxin n (%)	16 (29.1)
Nitrates n (%)	9 (16.4)

**Table 2:** Data of sleep study.

	**On diagnosis**	**On CPAP**	***P***
Total sleep time *(min)*	319.8 ± 74.1	330.1 ± 69.8	0.302
Apnea-hypopnea index *(no./h)*			
Total	45.3 ± 16.1	5.4 ± 4.1	<0.0001
Obstructive	38.5 ± 14.1	2.9 ± 2.5	<0.0001
Central	6.7 ± 7.3	2.4 ± 3.2	<0.0001
%TST SO_2_ <90% *(%)*	32.5 ± 33.6	2.8 ± 6.2	<0.0001
Lowest SO_2_ *(%)*	72.3 ± 16.2	87.4 ± 5.2	<0.0001
Arousal index *no./h*	43.9 ± 19.6	15.7 ± 10.3	<0.0001
Sleep Stage (% of TST)			
N1+2 *(%)*	82.5 ± 9.9	63.3 ± 13.5	<0.0001
N3 *(%)*	7.1 ± 6.9	17.6 ± 11.2	<0.0001
REM sleep *(%)*	10.4 ± 6.1	19.1 ± 7.1	<0.0001

Univariate regression analysis showed that age, BMI, atrial fibrillation, lowest SpO_2_, and pressure levels of CPAP were significantly associated with improvements in LVEF (Table 3), whereas the changes in variables after CPAP therapy, including BMI, BP, HR, norepinephrine concentration, and ESS, were not associated (Table 4). After adjusting for confounding variables, the age and BMI remained significant determinants of LVEF improvement (Table 5).

**Table 3:** Univariate regression analysis for baseline variables associated with the improvement of LVEF.

	**B**	**SE**	**β**	**T**	***P***
Age, per 1 year	−0.002	0.001	−0.454	−3.712	<0.001
Male Gender	0.032	0.027	0.159	1.173	0.246
Body mass index, per SD (5.4 kg/m^2^)	0.030	0.005	0.649	6.217	<0.001
Systolic blood pressure, per SD (13.3 mmHg)	0.008	0.006	0.167	1.236	0.222
Diastolic blood pressure, per SD (10.5 mmHg)	0.009	0.006	0.205	1.527	0.133
Heart rate, per SD (11.2 bpm)	−0.001	0.006	−0.006	−0.046	0.963
Left ventricular ejection fraction, per SD (9.8%)	0.104	0.006	0.229	1.710	0.0931
Norepinephrine, per SD (0.16 ng/ml)	−0.001	0.001	−0.121	−0.891	0.377
NYHA class III	−0.0207	0.012	−0.228	−1.706	0.0938
Ischemic heart disease	−0.0012	0.015	−0.011	−0.082	0.935
Atrial fibrillation	−0.0269	0.013	−0.272	−2.060	0.0443
ACE Inhibitors / AR blockers use	0.0294	0.016	0.243	1.821	0.0743
Beta blockers use	−0.0059	0.013	−0.065	−0.472	0.639
Diuretics use	0.0066	0.017	0.052	0.377	0.708
Spironoractone use	−0.0011	0.015	−0.010	−0.074	0.941
Nitrates use	−0.0074	0.017	−0.061	−0.442	0.660
Digoxin use	−0.0234	0.013	−0.237	−1.772	0.0821
Epworth sleepiness scale, per 1 point	0.0018	0.002	0.144	1.058	0.295
Apnea–hypopnea index, per SD (16.1 /h)	0.006	0.006	0.130	0.952	0.345
Obstructive apnea–hypopnea index, per SD (14.1 /h)	0.001	0.006	0.237	1.779	0.081
Central apnea–hypopnea index, per SD (7.3 /h)	−0.008	0.006	−0.171	−1.267	0.211
%TST SO_2_ <90%, per SD (33.6 %)	0.012	0.006	0.254	1.914	0.0611
Lowest SO_2_, per SD (16.2 %)	−0.004	0.006	−0.299	−2.280	0.0266
Arousal index, per SD (19.6 /h)	0.008	0.006	0.179	1.324	0.191
Slow wave sleep, per SD (6.9 %)	−0.001	0.006	−0.025	−0.184	0.855
REM sleep, per SD (6.1 %)	0.001	0.006	0.013	0.094	0.926
Pressure level, per 1cmH_2_O	0.009	0.003	0.423	3.400	0.0013

**Table 4:** The association of change of the variables and LVEF improvement.

	**B**	**SE**	**β**	**T**	***P***
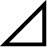 Body mass index,per SD (0.9 kg/m^2^)	0.006	0.007	0.130	0.954	0.344
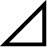 Systolic blood pressure, per SD (7.1mmHg)	−0.0004	0.006	−0.009	−0.064	0.949
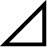 Diastolic blood pressure, per SD (6.6 mmHg)	−0.0008	0.006	−0.018	−0.128	0.899
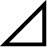 Heart rate, per SD (8.6 bpm)	−0.0007	0.006	−0.016	−0.117	0.907
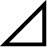 Norepinephrine, per SD (0.07 ng/ml)	−0.0002	0.001	−0.052	−0.380	0.706
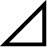 Epworth sleepiness scale, per 1 point	0.0018	0.004	0.065	0.477	0.636

**Table 5:** Multiple regression analysis for identify variables associated with LVEF improvement.

	**B**	**SE**	**B**	**T**	***P***
Age, per 1 year	−0.001	0.001	−0.284	−2.752	0.008
Body mass index, per SD (5.4 kg/m2)	0.005	0.001	−0.564	5.456	<0.001

## Discussion

In this study, 1-month CPAP treatment for OSA significantly increased LVEF improvements in Japanese patients with HF complicated by OSA. The degree of improvement in the LVEF was 6% in this population. Multivariate regression analyses showed that age and BMI were determinants of LVEF improvement.

Previous studies have shown that the degree of LVEF improvement by CPAP therapy varies from 5 to 9% (12, 13), which is consistent with our results. Kaneko et al. (12) examined the effects of CPAP on cardiac function in patients with reduced LVEF and OSA. They reported that CPAP therapy improved OSA, reduced daytime systolic BP and HR, and increased LVEF by 9%.

To date, several underlying mechanisms of how CPAP improves LVEF have been described. First, the obstructive apnea due to upper airway obstruction activates the sympathetic nervous system in patients with HF *via* hypoxia, hypercapnia, decreased cardiac output, and arousal from sleep (19, 20). Additionally, the acute arousal from sleep evokes sympathetic activation and BP elevation, which are carried over into the daytime (21–23). The treatment of OSA with CPAP therapy lowers sympathetic nervous overactivation, resulting in decreased BP and HR (23). The mechanisms of these effects remain uncertain but may be related to the adaptation of chemoreceptor reflexes or central processes governing autonomic outflow. Second, the respiratory efforts during obstructive apnea decrease the intrathoracic pressure, leading to increased left ventricular transmural pressure, left ventricular afterload, and cardiac metabolic demand (24, 25). Furthermore, augmentation of venous return to the right ventricle results in a leftward septal shift and reduced left ventricular preload and stroke volume (26). The CPAP therapy for exaggerated intrathoracic negative pressure may decrease left ventricular transmural pressure and left ventricular afterload, potentially counteracting or minimizing the effects of respiratory efforts during obstructive apnea on left ventricular systolic function (27–29). Meanwhile, the CPAP therapy increases intrathoracic pressure, which decreases systemic venous return, while positive pressure ventilation increases pulmonary vascular resistance and right ventricular afterload, thus, reducing the right ventricular stroke volume (30, 31), left ventricular preload, and stroke volume (32–35). These ambivalent effects of CPAP on hemodynamics may depend on an individual's specific preload and afterload states (30).

To date, the factors associated with LVEF improvement remain unknown. The novelty of our study is that we demonstrated age and BMI as determinants of LVEF improvement by CPAP. The LVEF was more likely to improve in young patients with obesity after 1 month of CPAP therapy.

Several speculations can be made regarding the mechanisms of these results. In patients with obesity, the pharyngeal fat deposition facilitates upper airway narrowing and collapses by increasing the external peripharyngeal soft tissue pressure. In addition, fat deposition to the chest wall leads to a reduction in compliance and an increase in airway resistance (36–38). An improvement in exaggerated negative intrathoracic pressure by CPAP is speculated to have a positive effect on hemodynamics more in patients with obesity, which may partly explain our finding that LVEF was more likely to improve in patients with obesity. Age is a determinant of the prognosis of HF, as shown in previous reports (3, 39–41). Advancing age is accompanied by the loss of cardiomyocytes *via* apoptosis and necrosis (42–44) and decreased responses to both sympathetic and parasympathetic antagonists (45). Based on these data, the elderly patients with HF might be less likely to show LVEF improvement with CPAP therapy for OSA.

### Limitations

This was a single-center, non-randomized study with a relatively small study population. Our data should be interpreted carefully, and further studies with larger populations are required to confirm our data. Another limitation is that one of the inclusion criteria was good adherence to CPAP therapy. Therefore, our results may not be generalizable. Regarding the lowest SpO2 on CPAP therapy of 87.4%, the residual hypoxia may affect the changes in LVEF, although detailed data of the sleep study on CPAP were unavailable. The LVEF measurement might be inaccurate in patients with atrial fibrillation, which may have affected the results of changes in LVEF, although LVEF was measured with a conventional averaging of three beats. In addition, we could not demonstrate an association between obesity and intrathoracic negative pressure because the intrathoracic pressure in the study population was not measured.

## Conclusions

Among patients with OSA and HF, the LVEF significantly improved after 1 month of CPAP therapy. The degree of LVEF improvement was significantly associated with age, BMI, atrial fibrillation, the severity of hypoxia, and the treatment pressure level of CPAP. After adjustment for baseline variables and changes in variables at 1 month, the LVEF was more likely to be improved in young patients with obesity.

## Data Availability Statement

The original contributions presented in the study are included in the article/supplementary material, further inquiries can be directed to the corresponding author.

## Ethics Statement

Ethical review and approval was not required for the study on human participants in accordance with the local legislation and institutional requirements. The patients/participants provided their written informed consent to participate in this study.

## Author Contributions

TK contributed to conception and design of the study. RN wrote the first draft of the manuscript. TD, HT, KN, and S-iM reviewed the manuscript. All authors contributed to manuscript revision, read, and approved the submitted version.

## Funding

Grant to the Intractable Respiratory Diseases and Pulmonary Hypertension Research Group, from the Ministry of Health, Labor, and Welfare, Japan Grant/Award No: 20FC1027; JSPS KAKENHI, Grant/Award No: JP17K09527, 21K08116.

## Conflict of Interest

RN and TK are affiliated with a department endowed by Philips Respironics, ResMed, and Fukuda Denshi. The remaining authors declare that the research was conducted in the absence of any commercial or financial relationships that could be construed as a potential conflict of interest.

## Publisher's Note

All claims expressed in this article are solely those of the authors and do not necessarily represent those of their affiliated organizations, or those of the publisher, the editors and the reviewers. Any product that may be evaluated in this article, or claim that may be made by its manufacturer, is not guaranteed or endorsed by the publisher.
